# Speech and language disorders in children from public schools in Belo Horizonte

**DOI:** 10.1016/j.rpped.2015.02.004

**Published:** 2015

**Authors:** Alessandra Terra Vasconcelos Rabelo, Fernanda Rodrigues Campos, Clarice Passos Friche, Bárbara Suelen Vasconcelos da Silva, Amélia Augusta de Lima Friche, Claudia Regina Lindgren Alves, Lúcia Maria Horta de Figueiredo Goulart

**Affiliations:** a Universidade Federal de Minas Gerais (UFMG), Belo Horizonte, MG, Brazil

**Keywords:** Speech, language and hearing sciences, School health, Speech, Language disorders

## Abstract

**Objective:**

To investigate the prevalence of oral language, orofacial motor skill and auditory processing disorders in children aged 4-10 years and verify their association with age and gender.

**Methods:**

Cross-sectional study with stratified, random sample consisting of 539 students. The evaluation consisted of three protocols: orofacial motor skill protocol, adapted from the Myofunctional Evaluation Guidelines; the Child Language Test ABFW - Phonology; and a simplified auditory processing evaluation. Descriptive and associative statistical analyses were performed using Epi Info software, release 6.04. Chi-square test was applied to compare proportion of events and analysis of variance was used to compare mean values. Significance was set at *p*≤0.05.

**Results:**

Of the studied subjects, 50.1% had at least one of the assessed disorders; of those, 33.6% had oral language disorder, 17.1% had orofacial motor skill impairment, and 27.3% had auditory processing disorder. There were significant associations between auditory processing skills’ impairment, oral language impairment and age, suggesting a decrease in the number of disorders with increasing age. Similarly, the variable "one or more speech, language and hearing disorders" was also associated with age.

**Conclusions:**

The prevalence of speech, language and hearing disorders in children was high, indicating the need for research and public health efforts to cope with this problem.

## Introduction

The need to communicate is inherent to human beings and is essential for the integral development, knowledge acquisition and learning. Language development involves physical, neurological, behavioral, cognitive, social and emotional aspects.

The adequacy of the auditory processing, which is the transformation of the acoustic signal into a meaningful message,^1^ is essential in language acquisition. Another important aspect is related to the orofacial motricity, which is associated with structural and functional aspects of the orofacial and cervical regions, including the functions of sucking, swallowing, chewing, breathing and articulation.^2^ For the oral language to occur, the sounds produced in the vocal folds are modeled and articulated during their passage through the larynx, pharynx, and oral and nasal cavities. It is necessary for the physical movements involved in the emission of sounds (phonetic aspects) to be produced adequately, while respecting the organizational aspects of the language sound system (phonoaudiological aspects).^3^

Some delays may occur, during child development that will have an adverse impact on children's lives. Phonoaudiological alterations may be responsible for these delays, leading to social maladjustments, learning and interpersonal skills’ difficulties, with negative effects on the overall development.^4^ Additionally, during the process of acquiring literacy, children transfer errors from the oral signs’ system to the written language,^5^ with learning difficulties being one of the main impacts of oral language alterations. Early recognition of these disorders, followed by appropriate interventions, can reduce the impact of these alterations on the lives of the affected children, allowing their social development and improving their quality of life.

The impact of communication disorders and evidence that the prevalence is high in schoolchildren^3^^,^ 4 ^,^ 6 ^-^ 11 justify further studies on the subject. Based on these data, it may be possible to develop effective actions for health promotion, speech therapy and pediatric interventions, which will help to prevent learning, emotional and social disorders.

This study aims to assess the prevalence of oral language, orofacial motricity and auditory processing alterations in children aged 4-10 years from public schools located in the area of a health care center in Belo Horizonte, as well as to verify their association with age and gender.

## Method

This is a cross-sectional study, approved by the Institutional Review Board of the Federal University of Minas Gerais (ETIC notice 263/08), with a representative random sample of 1853 children aged 4-10 years enrolled in the six public schools from the area served by the Health Care Center in the northeast region of Belo Horizonte. This health care center is a teaching, research and continuing education facility for students of the Universidade Federal de Minas Gerais, and it was used as a reference support facility for the referral of the assessed children.

Sample calculation considered as 40% the prevalence of phonoaudiological alterations,^3^^,^ 4 ^,^ 6 ^,^ 11 with a margin of error of 5%, 95% confidence interval, and increase of 15% for losses, resulting in 545 children, stratified by school and age range. Exclusion criteria were the presence of physical or cognitive limitations that prevented the tests to be performed, refusal to participate in the study and failure to obtain the Informed Consent form signed by a parent or child's legal guardian. A total of 539 children were assessed, with 1.1% of losses.

Each child was assessed by one of the 3 speech therapists specially trained on the evaluation criteria. Data collection was performed in three steps: orofacial motricity (OM) evaluation, oral language assessment and simplified auditory processing evaluation.

To assess orofacial motricity, a protocol developed by the researchers was used to assess the myofunctional aspects of the stomatognathic system, adapted from the Myofunctional Assessment Protocol.^2^ The protocol does not suggest rigid standards of normality, and alterations are defined on a case-by-case basis through observation by the speech therapist, based on his/her clinical experience. In this study, the criteria for any disorder were defined prior to the assessment by consensus of four speech therapists, considering morphological alterations, decreased tension and alterations in the movement of orofacial structures, and possible functional implications. The morphological aspects of the face, lips, tongue, and cheeks, and occlusion, tension and mobility of the lips, tongue and cheeks were evaluated by clinical observation; counter-resistance tests with disposable tongue depressor and gloved finger for verification of tension; puckering-smile movements, inflation and contraction of cheeks, protrusion and retraction of the tongue and movement of the tongue toward the four cardinal points to assess mobility.

The assessment of oral language was performed using the Child Language Test ABFW - Phonology,^12^ which consists in naming and imitation, in which the child's speech is recorded through phonetic transcription done during test application for further analysis. The test considers the age of seven as a marker for the end of the acquisition of phonetic and phonoaudiological aspects of language, in addition to defining what is expected for each age group.

We chose to use the term oral language when the following aspects were treated jointly: phonetic deviations, expressed by alterations in sound articulation, and phonological disorders, defined as language disorders characterized by the presence of productive asynchronous phonoaudiological processes (at an older range age than that when this same process is overcome by most children), and/or the presence of unusual phonoaudiological processes (which are not observed in the normal acquisition of the phonoaudiological system) in the child's speech.

The simplified evaluation of auditory processing^1^^,^ 13 involved the sequential memory to non-verbal sounds (SMNV), the sequential memory to verbal sounds (SMV) and the sound location test (SLT). Given the impossibility to assess pure tone thresholds through pure tone audiometry, we chose to assess the Cochleo-palpebral reflex (CPR) to exclude children with moderate or severe hearing loss. The criteria of test application and analysis of results complied with the rules defined by the test.^1^^,^ 13

Evaluations lasted approximately 30 min and were carried out in the school environment. The results were classified as normal or altered, being considered altered when the children had insufficient results in one or more tests, and normal when they achieved the requirements of the three tests. Children with altered tests were referred to speech therapy and pediatric care.

An electronic database was created, and Epi Info software, release 3.5.3, was used. The analysis of the frequency distribution of categorical variables was performed. The chi-square test was used to study the association between the presence of phonoaudiological alterations and gender. To verify the association between the presence of phonoaudiological alterations and age, the chi-square test for linear trend, the calculation of odds ratio and confidence intervals at 95% were used. A *p* value≤0.05 was considered statistically significant.

It is noteworthy that two other articles have already been published using the same sample of the present study, which have speech (phonetic and phonoaudiological disorders) as the main object, with one study being carried out in schoolchildren^14^ and one in preschool children.^15^ This article differs from the previous ones not only due to the larger sample size and broader age range, but also due to the objective, type of analysis and results shown. It should be noted that population-based studies that address the prevalence of phonoaudiological disorders are relatively scarce in the Brazilian literature, and the initiative to write this article also intended to fill this gap.

## Results

The distribution of the children according to age, gender and school is shown in Table 1. Of the evaluated children, 50.1% had at least one of the studied alterations. Oral language alterations were observed in 33.6% of the sample, followed by auditory processing (AP) disorders with 27.3% and orofacial motricity alterations in 17.1% (Table 2).

**Table 1:** Characteristics of the study population in the 2009/2010 period, Belo Horizonte (*n*=539).

Characteristics	*n*	%
*Age range*		
<5 years	67	12.4
5 to <7 years	185	34.3
7 to ≤11 years	287	53.3

*Gender*		
Male	287	53.2
Female	252	46.8

*School*		
School 1	84	15.6
School 2	98	18.2
School 3	114	21.1
School 4	36	6.7
School 5	21	3.9
School 6	186	34.5

*Total*	539	100.0

**Table 2:** Prevalence of phonoaudiological disorders (*n*=539).

Phonoaudiological disorder	*n*	%
Oral language	181	33.6
Phonological disorder	61	11.3
Phonetic disorder	93	17.3
Phonological + phonetic disorder	27	5.0
Orofacial motricity	92	17.1
Auditory processing^a^	147	27.3
Children with 1 or more alterations^b^	270	50.1

a The categories are not mutually exclusive. The child may have more than one type of alteration.

b Six children were excluded due to inconclusive AP test.

In the age group 7-10 years linguistic variation was observed, in which the simplification of consonant cluster and final consonant simplification appeared in specific test words in 38.5% (*n*=111) of the children. These cases were analyzed separately and were not considered alterations, but linguistic variations, because they are regional characteristics of speech. Children younger than 7 years employ these phonoaudiological processes, according to the analysis of the Child Language Test ABFW - Phonology.^12^For this reason, in this age group these same alterations were not considered linguistic variations, but phonoaudiological processes.

The most commonly found speech disorder was simplification - the final consonant and consonant cluster simplification - which occurred in 36.4% of children with oral language alteration. The main phonetic alteration was the frontal lisp, present in 30.4% of children with oral language alteration.

The tests of sequential memory to non-verbal and verbal sounds were altered in 56.9% and 49.0% of children with auditory processing disorders, respectively. In children with altered orofacial motricity, 72.1% of these changes were related to the positioning of the articulatory organs, and in 67.4%, to the tension of the assessed structures.

There was an association between the children's age group and the presence of one or more assessed phonoaudiological disorders, oral language and auditory processing alterations (Table 3). The chance of a child younger than five years to have one or more phonoaudiological alterations was 2.49 times higher (95%CI: 1.37-4.53) than the chance of a child aged ≥7 years. The same was not observed for children aged 5-7 years. Similarly, younger children were twice as likely (OR=2.15; 95%CI 1.21-3.81) to have oral language alterations than older ones, but there was no difference if compared to those age 5-7 years. The Odds Ratio of children younger than 7 years to have auditory processing disorders, when compared with those aged >7 years was 9.72 (95%CI: 5.06-18.81) for the group aged <5 years, and 1.92 (95%CI: 1.21-3.05) for those aged 5-7 years. There was no association between age range and orofacial motricity alterations, or between gender and the assessed phonoaudiological disorders.

**Table 3:** Distribution of phonoaudiological disorders in relation to age range and gender (%).

	Age range in years (%)^a^				Gender (%)^b^		
	<5	5 to <7	7 to <11		Male		Female
	(*n*=67)	(*n*=185)	(*n*=287)		(*n*=287)		(*n*=252)
*One or more alterations*							
Yes (*n*=270)	67.2	51.9	45.1		53.0		47.0
No (*n*=269)	32.8	48.1	54.9		53.5		46.5
*p*value						0.96	
OR	2.49	1.31	1				
95%CI	1.37–4.53	0.87–1.94	–				

*Oral language alterations*							
Yes (*n*=181)	50.7	29.2	32.4		50.3		49.7
No (*n*=358)	49.3	70.8	67.6		54.7		45.3
*p*value						0.37	
OR	2.15	0.86	1				
95%CI	1.21–3.81	0.56–1.31	–				

*OM alterations*							
Yes (*n*=92)	16.4	18.4	16.4		59.8		40.2
No (*n*= 447)	83.6	81.6	83.6		51.9		48.1
*p*value						0.20	
OR	1.0	1.15	1				
95%CI	0.46–2.16	0.69–1.92	–				

*AP alterations* c							
Yes (*n*= 147)	67.7	29.3	17.8		51.0		49.0
No (*n*= 386)	32.3	70.6	82.2		53.6		46.4
*p*value						0.66	
OR	9.72	1.92	1				
95%CI	5.06–18.81	1.21–3.05	–				

OR, odds ratio; OM, orofacial motricity; AP, auditory processing.

a Chi-square test for linear trend.

b Chi-square.

c 6 children showed an inconclusive result and were not included in the analysis.

## Discussion

Studies on the prevalence of phonoaudiological alterations, especially those addressing more than one type of alteration in a single population, are scarce in the literature. This study showed a high prevalence of some types of alterations in a population of children aged 4-10 years. Validated, yet simple and easy-to-apply tests were used, eliminating the need for sophisticated equipment. Despite these advantages, the high sensitivity of these tests, carried out alone, may have led to a possible overestimation of the prevalence of alterations. Another aspect to be considered is that some children may have shown transient alterations during their development. However, due to the cross-sectional characteristic of the study, which did not aim to follow the children longitudinally, we chose to use the criteria indicated by the tests to classify alterations at the time of assessment.

The prevalence of oral language disorders indicated by this study is between 21% and 49%, within the same range found in studies carried out in Brazil.^6^^,^ 8 ^,^ 16 ^-^ 20 This variation is probably due to methodological differences, economic factors and age of the children included in the samples, making it difficult to compare them. The international studies indicate a lower prevalence of alterations.^21^^,^ 22 In the Cuban population, a study found a 12% prevalence of oral language disorders,^21^ whereas in Australia,^22^ 13% of the children showed results below the expected average for this age range.

The literature indicates that the prevalence of phonological disorders varies from 9.2% to 18.6%,^5^^,^ 8 ^,^ 23 values that are close to those found in our study. The prevalence of phonetic alterations ranges from 2.1% to 22.5% in different regions of Brazil.^8^^,^ 23 ^,^ 24 A previous study found a prevalence of 22.5% of a type of phonetic deviation (lisp).^24^ In Montes Claros (MG), the lisp appeared in 8.4% of 404 children with a mean age of 6 years and 5 months.^19^ In the present study, among the phonetic alterations, the lisp was also the most prevalent disorder. In another study with children from private schools, the results showed a prevalence of 18% of phonetic alterations, using a different type of assessment than that used in this study.^23^ Although the results are similar, to those found in this study, the use of different tools makes it difficult to compare it with results from other studies.

Regarding the linguistic variation found, it was observed that the speech of individuals living in the city of Belo Horizonte, where this research was performed, included the same variations that often occur in the speech of adults.^25^ This fact may explain the occurrence of the same simplifications in the children's speech production, as children tend to follow the speech pattern of their social group. A study carried out in Belo Horizonte showed that children of mothers who had the consonant cluster simplification tended to repeat these simplifications in their speech.^25^

The prevalence of failure in the simplified auditory processing evaluation described in the literature is between 24.6% and 44%.^10^^,^ 11 ^,^ 26 In this study, the failure to respond to auditory processing assessment test is close to that observed in another study that used the same tool.^11^ In a study carried out with schoolchildren, using simplified auditory processing assessment, tympanometry and acoustic reflex evaluation, it was observed that most children (64%) who failed the test were the youngest in the sample.^26^These results are similar to those of the present study.

In the simplified auditory processing evaluation, more difficulty was observed in tests that assessed the auditory capacity of simple temporal ordering, i.e., identification of successive acoustic events, which are the tests of Sequential Memory to Verbal and Nonverbal Sounds, than discrimination of the sound source direction, which is the location test. The short-term auditory memory, involved in temporal ordering, is an important skill for reading and writing, as, to perform these tasks, one must store the contents to go forward. The same difficulty was observed in the literature.^26^ Among the simple temporal ordering skills, poorer performance was observed in the test of sequential memory to non-verbal sounds, corroborating another study.^27^

When used together, the three procedures used for the simplified auditory processing assessment (sound localization, sequential memory to verbal and nonverbal sounds) have a sensitivity of 80%, i.e., they are effective in identifying the auditory processing disorder among the ones who have it. Failure in one of the tests of the simplified auditory processing assessment is indicative of inadequacy and, in these cases, one should refer the child to complete auditory processing assessment to confirm the diagnosis.

The simplified auditory processing assessment was developed to be carried out in diotic listening conditions, in which the same stimulus is offered simultaneously to both ears, and the medical literature recommends that it should be performed in a quiet environment.^1^ In this study, it was not possible to measure the noise level using a sound pressure level device. Aiming to minimize a possible interference by noise, the evaluators performed the tests in a quiet room, with only the presence of the children and the evaluator, at different times from the break interval between classes. Nevertheless, one cannot rule out the possibility of interference of the environment where the simplified auditory processing evaluations were performed, which may have led to overestimation of the results. The presence of noise generates loss of concentration and attention, which may increase the number of errors, decreasing the quality of the daily tasks. In a previous study using the auditory processing tests in diotic listening of tonal pattern of frequency and duration, it was observed that subjects with and without phonoaudiological alterations showed worse performance in the presence of noise.^28^

It was verified that orofacial motricity aspects are under-researched in apparently healthy children with no complaints. Usually, the assessed children have other types of previously diagnosed phonoaudiological, dental or respiratory alterations. A study of 50 apparently healthy children aged 5-8 years found a prevalence of 84.0% of orofacial motricity alterations,^29^ a much higher value than that found in this study. This discrepancy can be attributed to differences in sample size and composition. Moreover, in the abovementioned study, the use of a questionnaire showed that most of the children had an inadequate dietary transition, a pacifier habit, and mastication, swallowing, breathing and occlusion alterations, which may have facilitated the occurrence of orofacial motricity disorders. Another study showed that 32.5% of the 1103 children assessed through phonoaudiological screening in 15 municipal elementary schools of Vila Velha (ES) showed alterations in orofacial motricity, a higher percentage than that found in the present study.^18^ In a town located in the countryside of the state of Paraná, 77.5% of 31 children from a public school had orofacial motricity alterations.^30^ In a study carried out in the state of Ceará, orofacial motricity alterations were divided by the affected structures, with prevalences of 27% in the cheeks, 18% in the lips and 7% in the tongue.^20^

Although some studies^6^^,^ 8 ^,^ 19 have shown a higher prevalence of phonoaudiological alterations in males, especially in relation to phonological disorders, this finding was not confirmed in this study.

The associations observed between the presence of one or more phonoaudiological disorders, the auditory processing alterations and oral language alterations with age range can be explained by the gradual development of children during normal neuropsychomotor maturation. These associations indicate that younger children, especially children younger than 5 years, who are still acquiring communication skills, can have transient manifestations, which do not necessarily characterize phonoaudiological disorders.

On the other hand, it is known that children diagnosed with alterations after 7 years of age already had indications of changes in the early stages of development, which emphasizes the importance of phonoaudiological assessments in younger children. Additionally, it is observed that some assessed children had more than one simultaneous phonoaudiological disorder. This finding reiterates the importance of detection of these disorders on a timely manner. An early diagnosis can prevent deterioration of the initial condition, as one alteration may be associated with the other.^14^

This study is not without limitations, with the first one being its cross-sectional characteristic, without longitudinal follow-up of the children and no inferences about causality. The impossibility of performing otoscopy and audiometry before the auditory processing assessment and the environmental conditions for carrying out the tests are also recognized as limitations. Nevertheless, this study contributes to the advancement of knowledge by addressing different alterations in a representative sample of a population of children with no previous complaints.

The high percentage of children with oral language, orofacial motricity and auditory processing alterations found in the study population may be indicative of the prevalence of these disorders in other populations with similar characteristics. The results of this study emphasize the need for research and actions in communication health to face this problem, and further reinforce the need to train health and education professionals for the timely detection of children with possible phonoaudiological alterations. We expect the results of this research may help in the planning of intersectorial and multidisciplinary, educational and/or assistance actions that will offer the opportunity for adequate child development and overall communication health promotion.
