# Supplementary material for: Speech and language disorders in children from public schools in Belo Horizonte
Source: Rev Paul Pediatr. 2015 Oct-Dec;33(4):453–9. doi: 10.1016/j.rpped.2015.02.004 (PMC4685566; doi:10.1016/j.rpped.2015.02.004)
Supplement: Translation

# Translation: Alterações fonoaudiológicas em crianças de escolas públicas em Belo Horizonte

**Authors:** Alessandra Terra Vasconcelos Rabelo, Fernanda Rodrigues Campos, Clarice Passos Friche, Bárbara Suelen Vasconcelos da Silva, Amélia Augusta de Lima Friche, Claudia Regina Lindgren Alves, Lúcia Maria Horta de Figueiredo Goulart

**Affiliations:** a Universidade Federal de Minas Gerais (UFMG), Belo Horizonte, MG, Brasil

**Keywords:** Fonoaudiologia, Saúde escolar, Fala, Transtornos da linguagem

## Abstract

**Objetivo:**

Investigar a prevalência de alterações de linguagem oral, motricidade orofacial e processamento auditivo em crianças de 4-10 anos e verificar a sua associação com a idade e o gênero.

**Métodos:**

Estudo transversal com amostra aleatória e estratificada, composta por 539 crianças. Foram usados para avaliação protocolo de motricidade orofacial, adaptado do Roteiro para Avaliação Miofuncional; prova de Fonologia do Teste de Linguagem Infantil ABFW; e avaliação simplificada do processamento auditivo. Foram feitas análises estatísticas descritivas e de associação com o *software* Epi Info, versão 6.04. Para comparar as proporções foi empregado o qui-quadrado e para comparar médias foi empregada a análise de variância. Foi considerado significativo *p*≤0,05.

**Resultados:**

Das crianças avaliadas, 50,1% apresentaram pelo menos uma das alterações estudadas, 33,6% mostraram alteração de linguagem oral, 17,1% de motricidade orofacial e 27,3% do processamento auditivo. Observou-se associação significativa entre alterações fonoaudiológicas de processamento auditivo, linguagem oral e a faixa etária, o que sugere diminuição do número de crianças com alterações fonoaudiológicas com o aumento da idade. A variável "uma ou mais alterações fonoaudiológicas" também se associou à faixa etária, de maneira similar à acima descrita.

**Conclusões:**

A prevalência de alterações fonoaudiológicas na população estudada foi considerada alta, o que evidencia a necessidade de pesquisas e ações em saúde para o enfrentamento do problema.

## Introdução

A necessidade da comunicação é inerente ao ser humano e é essencial para o desenvolvimento integral, a aquisição de conhecimento e a aprendizagem. O desenvolvimento da linguagem envolve aspectos físicos, neurológicos, comportamentais, cognitivos, sociais e afetivos.

A adequação do processamento auditivo, que consiste na transformação do sinal acústico em mensagem com significado,^1^ é fundamental na aquisição da linguagem. Outro aspecto importante se refere à motricidade orofacial, que se relaciona aos aspectos estruturais e funcionais das regiões orofacial e cervical, incluindo funções de sucção, deglutição, mastigação, respiração e articulação.^2^Para que a linguagem oral ocorra, os sons produzidos nas pregas vocais são modelados e articulados na sua passagem pela laringe e faringe e pelas cavidades oral e nasal. É necessário que os movimentos físicos envolvidos na emissão dos sons (aspectos fonéticos) sejam produzidos de forma apropriada, bem como respeitados os aspectos organizacionais do sistema de sons da língua (aspectos fonológicos).^3^

Durante o desenvolvimento infantil podem ocorrer atrasos que repercutem desfavoravelmente na vida das crianças. Alterações fonoaudiológicas podem ser responsáveis por esses atrasos e levar a desajustes sociais, dificuldades escolares e de relacionamento interpessoal, com repercussões negativas no desenvolvimento global.^4^ Além disso, na alfabetização, as crianças transferem os erros do sistema de signos orais para o escrito.^5^ As dificuldades de aprendizagem são um dos principais impactos das alterações de linguagem oral. O reconhecimento precoce desses distúrbios, seguido de intervenções adequadas, pode reduzir os prejuízos na vida das crianças afetadas e possibilitar seu desenvolvimento social e sua melhor qualidade de vida.

Os impactos das desordens da comunicação e indicações de que a prevalência seja elevada em escolares^3^^,^ 4 ^,^ 6 ^-^ 11justificam o estudo mais amplo do tema. A partir desses dados, poderá ser possível elaborar ações efetivas de promoção de saúde e intervenção fonoaudiológica e pediátrica, que auxiliarão na prevenção de distúrbios de aprendizagem, emocionais e sociais.

Este trabalho tem como objetivo estudar a prevalência de alterações de linguagem oral, motricidade orofacial e processamento auditivo em crianças de quatro a dez anos de escolas públicas da área de abrangência de um centro de saúde de Belo Horizonte, bem como verificar a sua associação com idade e gênero.

## Método

Estudo transversal, aprovado pelo Comitê de Ética em Pesquisa da Universidade Federal de Minas Gerais (parecer Etic 263/08), com amostra aleatória representativa da população de 1.853 crianças de quatro a 10 anos matriculadas nas seis escolas públicas da área de abrangência de centro um de saúde da região nordeste de Belo Horizonte. Esse centro de saúde é cenário de ensino, pesquisa e extensão para estudantes da Universidade Federal de Minas Gerais e serviu de ponto de apoio para a pesquisa e referência para encaminhamento das crianças avaliadas.

O cálculo da amostra tomou como 40% a prevalência das alterações fonoaudiológicas,^3^^,^ 4 ^,^ 6 ^,^ 11margem de erro de 5%, intervalo de confiança de 95%, acréscimo de 15% de perdas. No total foram 545 crianças, estratificadas por escola e faixa etária. Foram critérios de exclusão a presença de limitações físicas ou cognitivas que impossibilitassem os testes, a não concordância em participar da pesquisa e a não apresentação do Termo de Consentimento Livre e Esclarecido assinado por um dos pais ou responsáveis. Foram avaliadas 539 crianças, com 1,1% de perdas.

Cada criança foi avaliada por uma fonoaudióloga. No total eram três avaliadoras devidamente treinadas quanto aos critérios. A coleta de dados foi feita em três etapas: avaliação de motricidade orofacial (MO), avaliação da linguagem oral e avaliação simplificada do processamento auditivo.

Para aferir a motricidade orofacial, foi usado protocolo elaborado pelas pesquisadoras a fim de avaliar aspectos miofuncionais do sistema estomatognático, adaptado do Roteiro para Avaliação Miofuncional.^2^ O protocolo não sugere padrões rígidos de normalidade. As alterações são definidas caso a caso pela observação do fonoaudiólogo, a partir de sua experiência clínica. Nesta pesquisa, os critérios para que a criança fosse considerada com alteração foram definidos antes das avaliações pelo consenso de quatro fonoaudiólogas, considerando alterações morfológicas, diminuição na tensão e alteração na movimentação das estruturas orofaciais, além de possíveis repercussões funcionais. Os aspectos morfológicos de face, lábios, língua, bochechas e oclusão, tensão e mobilidade de lábios, língua e bochechas foram avaliados por meio de observação clínica; testes de contrarresistência com espátula de madeira descartável e dedo enluvado para a verificação de tensão; movimentos de bico-sorriso, inflação e contração de bochechas, protrusão e retração de língua e movimento da língua em direção aos quatro pontos cardeais para a verificação da mobilidade.

A avaliação da linguagem oral usou o Teste de Avaliação de Linguagem - ABFW - Fonologia,^12^ que consiste em citação e imitação, nas quais a fala da criança é anotada por meio de transcrição fonética feita durante a aplicação, para posterior análise. O teste considera a idade de sete anos como marcador do fim da aquisição dos aspectos fonéticos e fonológicos de linguagem, além de definir o que é esperado para cada faixa etária.

Optou-se pelo uso do termo linguagem oral quando os seguintes aspectos foram tratados de maneira conjunta: desvios fonéticos, caracterizados pelas alterações de articulação dos sons, e desvios fonológicos, definidos como alterações de linguagem caracterizadas pela presença de processos fonológicos produtivos assincrônicos (em faixa etária superior àquela de superação desse mesmo processo pela maior parte das crianças) e/ou pela presença de processos fonológicos incomuns (que não são observados na aquisição normal do sistema fonológico) na fala da criança.

A avaliação simplificada do processamento auditivo^1^^,^ 13 envolveu o Teste de Memória Sequencial para Sons Não Verbais (MSNV), o Teste de Memória Sequencial para Sons Verbais (MSV) e o Teste de Localização Sonora (LS). Diante da impossibilidade de avaliação dos limiares tonais por meio de audiometria tonal limiar, optou-se pela pesquisa do Reflexo Cócleo-Palpebral (RCP) para a exclusão das crianças com perda auditiva moderada ou severa. Os critérios de aplicação e análise dos resultados respeitaram as regras definidas pelo teste.^1^^,^ 13

As avaliações duraram aproximadamente 30 minutos e foram feitas no próprio ambiente escolar. Os resultados foram classificados em normais ou alterados e foram considerados alterados quando as crianças tiveram resultado insuficiente em uma ou mais provas e normais quando apresentaram os resultados exigidos nas três provas. As crianças com testes alterados foram encaminhadas para atendimento fonoaudiológico e pediátrico.

Foi constituído banco de dados eletrônico e usado o programa Epi Info, versão 3.5.3. Fez-se análise da distribuição de frequência das variáveis categóricas. Usou-se o teste do qui-quadrado para estudar a associação entre a presença de alterações fonoaudiológicas e gênero. Para verificar a associação entre a presença de alterações fonoaudiológicas e idade usou-se o teste de qui-quadrado de tendência linear e cálculo de *odds ratio* e intervalos de confiança a 95%. O *p*-valor≤0,05 foi considerado limiar de significância estatística.

Vale ressaltar que já foram publicados dois artigos que usaram a mesma casuística do presente estudo que têm a fala (desvios fonéticos e fonológicos) como principal objeto, um com escolares^14^ e outro com pré-escolares.^15^ O presente artigo difere dos anteriores não apenas pela ampliação da amostra e faixa etária, mas pelo objetivo, tipo de análise e pelos resultados apresentados. Ressalta-se que estudos de base populacional que abordem a prevalência de alterações fonoaudiológicas são relativamente escassos na literatura brasileira e a iniciativa de redação desse artigo teve também como objetivo suprir essa lacuna.

## Resultados

A distribuição das crianças avaliadas segundo faixa etária, gênero e escola é apresentada na Tabela 1. Das crianças avaliadas, 50,1% apresentaram pelo menos uma das alterações estudadas. Alterações da linguagem oral foram observadas em 33,6% da amostra, seguidas pelas alterações do processamento auditivo com 27,3% e motricidade orofacial em 17,1% (Tabela 2).

**Tabela 1:** Caracterização da população estudada quanto a faixa etária, gênero e escola de 2009/2010, Belo Horizonte (n=539)

Características	n	%
*Faixa etária*		
<5 anos	67	12,4
≥5 anos e <7 anos	185	34,3
≥7 anos e ≤10 anos	287	53,3

*Gênero*		
Masculino	287	53,2
Feminino	252	46,8

*Escola*		
Escola 1	84	15,6
Escola 2	98	18,2
Escola 3	114	21,1
Escola 4	36	6,7
Escola 5	21	3,9
Escola 6	186	34,5

*Total*	539	100

**Tabela 2:** Prevalência de alterações fonoaudiológicas (n=539)

Alteração fonoaudiológica	n	%
Linguagem oral	181	33,6
Desvio fonológico	61	11,3
Desvio fonético	93	17,3
Desvio fonológico + Desvio fonético	27	5,0
Motricidade orofacial	92	17,1
Processamento auditivo^a^	147	27,3
Crianças com 1 ou mais alterações^b^	270	50,1

a As categorias não são excludentes. A criança pode apresentar mais de um tipo de alteração.

b Excluídas seis crianças com teste de PA inconclusivo.

Na faixa etária de sete a 10 anos, foi observada variação linguística em que a simplificação do encontro consonantal e a simplificação da consoante final apareceram em palavras específicas do teste em 38,5% (n=111) das crianças. Esses casos foram analisados separadamente e não foram consideradas alterações, e sim variações linguísticas, por serem características regionais de fala. As crianças abaixo de sete anos empregam esses processos fonológicos, de acordo com a análise do teste de Fonologia do Teste de Linguagem Infantil - ABFW.^12^ Por esse motivo, nesse grupo essas mesmas trocas não foram consideradas variações linguísticas, e sim processos fonológicos.

O tipo de desvio fonológico mais encontrado foi o de simplificação - de consoante final e encontro consonantal - que ocorreu em 36,4% das crianças com alteração de linguagem oral. A principal alteração fonética foi o ceceio anterior, presente em 30,4% das crianças com alteração de linguagem oral.

As provas de memória sequencial não verbal e verbal estiveram alteradas em respectivamente 56,9% e 49,0% das crianças com desvios na avaliação do processamento auditivo. Nas crianças com motricidade orofacial alterada, 72,1% dessas alterações se relacionaram ao posicionamento dos órgãos articulatórios e 67,4% com a tensão das estruturas avaliadas.

Houve associação entre a faixa etária das crianças e a presença de uma ou mais alterações fonoaudiológicas estudadas, alterações de linguagem oral e de processamento auditivo (Tabela 3). A chance de uma criança com menos de cinco anos apresentar uma ou mais alterações fonoaudiológicas foi 2,49 (IC95% 1,37-4,53) vezes a chance de uma criança ≥7 anos. O mesmo não foi observado em relação às crianças de cinco a sete anos. Da mesma forma, as crianças mais jovens apresentaram duas vezes mais chance (OR=2,15; IC95% 1,21-3,81) de apresentar alterações de linguagem oral do que as mais velhas, porém sem diferença em relação às de cinco a sete anos. A chance de as crianças menores de sete anos apresentarem alterações do processamento auditivo, comparadas com as de mais de sete anos, foi de 9,72 (IC95% 5,06-18,81) para o grupo com idade menor do que cinco anos e de 1,92 (IC95% 1,21-3,05) para as de cinco a sete anos. Não houve associação entre faixa etária e alterações da motricidade orofacial e nem de gênero com as alterações fonoaudiológicas avaliadas.

**Tabela 3:** Distribuição percentual das alterações fonoaudiológicas em relação a faixa etária e gênero

	Faixa etária em anos (%)^a^				Gênero (%)^b^		
	<5 (n=67)	≥5 e <7 (n=185)	≥7 e ≤10 (n=287)		Masc. (n=287)		Fem. (n=252)
*Uma ou mais alterações*							
Sim (n=270)	67,2	51,9	45,1		53,0		47,0
Não (n=269)	32,8	48,1	54,9		53,5		46,5
*p*‐valor						0,96	
OR	2,49	1,31	1				
IC95%	1,37‐4,53	0,87‐1,94	–				

*Alterações de linguagem oral*							
Sim (n=181)	50,7	29,2	32,4		50,3		49,7
Não (n=358)	49,3	70,8	67,6		54,7		45,3
*p*‐valor						0,37	
OR	2,15	0,86	1				
IC95%	1,21‐3,81	0,56‐1,31	–				

*Alterações de MO*							
Sim (n=92)	16,4	18,4	16,4		59,8		40,2
Não (n=447)	83,6	81,6	83,6		51,9		48,1
*p*‐valor						0,20	
OR	1,0	1,15	1				
IC95%	0,46‐2,16	0,69‐1,92	–				

*Alterações de PA* c							
Sim (n=147)	67,7	29,3	17,8		51,0		49,0
Não (n=386)	32,3	70,6	82,2		53,6		46,4
*p*‐valor						0,66	
OR	9,72	1,92	1				
IC95%	5,06‐18,81	1,21‐3,05	–				

Masc., masculino; Fem., feminino; OR, *odds ratio*; MO, motricidade orofacial; PA, processamento auditivo.

a Qui‐quadrado de tendência linear.

b Qui‐quadrado.

c Seis crianças apresentaram resultado inconclusivo e não foram incluídas na análise.

## Discussão

Os estudos de prevalência de alterações fonoaudiológicas, especialmente os que abordam mais de um tipo de alteração em uma única população, são escassos na literatura. Este estudo mostrou a alta prevalência de alguns tipos de alterações em uma população de crianças de quatro a 10 anos. Foram usados testes validados, porém simples e de fácil aplicação, que dispensam o uso de equipamentos sofisticados. Apesar dessas vantagens, a alta sensibilidade desses testes, feitos de forma isolada, pode ter levado a possível superestimação da prevalência das alterações. Outro aspecto a ser considerado é que algumas crianças podem ter apresentado alterações transitórias do desenvolvimento. Entretanto, pela característica transversal do estudo, que não se propôs a acompanhar longitudinalmente as crianças, optou-se por usar os critérios indicados pelos testes para classificar as alterações no momento da avaliação.

A prevalência de alterações de linguagem oral apontada por este trabalho se encontra entre 21% a 49%, mesma faixa encontrada em estudos feitos no Brasil.^6^^,^ 8 ^,^ 16 ^-^ 20Essa variação provavelmente se deve a diferenças metodológicas, fatores econômicos e de idade das crianças que compuseram as amostras, o que dificulta a comparação entre eles. Já estudos internacionais apontam menor prevalência de alterações.^21^^,^ 22 Na população cubana, pesquisa apontou como 12% a prevalência de desordens na linguagem oral^21^ e na Austrália^22^13% das crianças avaliadas apresentaram resultado abaixo da média esperada para a faixa etária.

A literatura aponta que a prevalência de desvio fonológico varia de 9,2% a 18,6%,^5^^,^ 8 ^,^ 23 valores próximos aos encontrados neste estudo. Já a prevalência de desvios fonéticos varia de 2,1% a 22,5% em diferentes regiões do Brasil.^8^^,^ 23 ^,^ 24 Em estudo anterior foi verificada prevalência de 22,5% de um tipo de desvio fonético (ceceio).^24^Em Montes Claros (MG), o ceceio apareceu em 8,4% de 404 crianças com média de seis anos e cinco meses.^19^ No presente estudo, entre os desvios fonéticos, o ceceio também foi mais prevalente. Em outra pesquisa com crianças de escolas particulares, os resultados mostram prevalência de 18% de desvio fonético, com o uso de avaliação distinta da escolhida pela presente pesquisa.^23^Apesar da proximidade desses valores com os encontrados neste trabalho, o uso de diferentes instrumentos torna difícil a comparação com os resultados de outros estudos.

Em relação à variação linguística encontrada, observa-se que na fala de moradores de Belo Horizonte, onde foi feita esta pesquisa, as mesmas variações ocorrem frequentemente também na fala dos adultos.^25^ Esse fato pode explicar a ocorrência das mesmas simplificações nas produções de fala das crianças, pois essas tendem a aproximar sua fala do padrão do grupo social. Estudo feito em Belo Horizonte mostrou que filhos de mães que apresentavam simplificações do encontro consonantal tendiam a repetir essas simplificações nas suas falas.^25^

A prevalência de falha na avaliação simplificada do processamento auditivo descrita na literatura se situa entre 24,6% a 44%.^10^^,^ 11 ^,^ 26 Neste estudo, a falha na resposta ao teste de avaliação do processamento auditivo se aproxima do observado em outra pesquisa que usou o mesmo instrumento.^11^ Em pesquisa feita com escolares, com avaliação simplificada do processamento auditivo, timpanometria e verificação do reflexo acústico, observou-se que a maioria (64%) das crianças que falharam era constituída pelas mais jovens da amostra,^26^ resultado semelhante ao do presente estudo.

Na avaliação simplificada do processamento auditivo observaram-se mais dificuldade nas provas que testavam a habilidade auditiva de ordenação temporal simples, ou seja, identificação de eventos acústicos sucessivos, que são os testes de memória sequencial não verbal e verbal, do que discriminação da direção da fonte sonora, que é o teste de localização. A memória auditiva de curto prazo, envolvida na ordenação temporal, é uma habilidade importante para a leitura e escrita, uma vez que, para executar essas tarefas, é necessário armazenar o conteúdo para seguir adiante. A mesma dificuldade foi observada na literatura.^26^ Entre as habilidades de ordenação temporal simples, verificou-se pior desempenho no teste de memória sequencial para sons não verbais, o que corrobora outra pesquisa.^27^

Quando usados em conjunto, os três procedimentos da avaliação simplificada do processamento auditivo (localização sonora, memória sequencial para sons verbais e não verbais) apresentam sensibilidade de 80%, ou seja, são eficientes em identificar corretamente a desordem do processamento auditivo entre aqueles que a têm. A falha em uma das provas da avaliação simplificada do processamento auditivo é indicativa de inadequação e, nesses casos, deve-se encaminhar a criança para avaliação completa do processamento auditivo para confirmação do diagnóstico.

A avaliação simplificada do processamento auditivo foi desenvolvida para ser aplicada em escuta diótica, na qual estímulos iguais são apresentados simultaneamente para ambas as orelhas e é recomendação da literatura que seja feita em ambiente silencioso.^1^ Nesta pesquisa não foi possível medir o nível de ruído com o uso de um medidor de nível de pressão sonora. Com o intuito de minimizar uma possível interferência de ruído, foi tomado o cuidado de fazer os testes em sala silenciosa, com a presença apenas da criança e do avaliador, em horário diferente do intervalo entre aulas. Apesar disso, não se pode descartar a possibilidade de interferência do ambiente no qual foram feitas as avaliações simplificadas do processamento auditivo, o que pode ter levado a uma superestimação no resultado encontrado. A presença de ruído gera perda de concentração e atenção, o que pode aumentar o número de erros e diminuir a qualidade de tarefas cotidianas. Em estudo anterior, com os testes de processamento auditivo em escuta diótica de padrão tonal de frequência e duração, observou-se que sujeitos com e sem alterações fonoaudiológicas apresentaram pior desempenho na presença de ruído.^28^

Verificou-se que os aspectos de motricidade orofacial são pouco pesquisados em crianças aparentemente saudáveis e sem queixas. Geralmente as crianças avaliadas apresentam outros tipos de alterações fonoaudiológicas, dentárias ou respiratórias já diagnosticadas. Em estudo com 50 crianças de cinco a oito anos aparentemente saudáveis, verificou-se prevalência de 84% de alterações de motricidade orofacial,^29^ valor muito superior ao encontrado nesta pesquisa. Essa discrepância pode ser atribuída à diferença na composição e ao tamanho das amostras. Além disso, no estudo acima citado, o uso de um questionário evidenciou que grande parte das crianças havia tido transição alimentar inadequada, hábito de chupeta, alterações de mastigação, deglutição, respiração, oclusão, o que pode ter facilitado a ocorrência de distúrbios da motricidade orofacial. Já outro estudo mostrou que 32,5% das 1.103 crianças avaliadas por triagem fonoaudiológica em 15 escolas municipais de ensino fundamental de Vila Velha (ES) apresentaram alteração de motricidade orofacial, porcentagem também maior do que a encontrada na presente pesquisa.^18^ Em uma cidade do interior do Paraná, 77,5% de 31 crianças de escola pública apresentaram alteração da motricidade orofacial.^30^ Já em estudo feito no Ceará, as alterações de motricidade orofacial foram divididas pelas estruturas e suas prevalências foram de 27% (bochechas), 18% (lábios) e 7% (língua).^20^

Embora alguns estudos^6^^,^ 8 ^,^ 19 tenham mostrado a maior prevalência de alterações fonoaudiológicas no gênero masculino, principalmente em relação ao desvio fonológico, esse achado não se confirmou nesta pesquisa.

As associações encontradas entre a presença de uma ou mais alterações fonoaudiológicas, as alterações de processamento auditivo e as de linguagem oral com a faixa etária podem ser explicadas pelo desenvolvimento gradual das crianças durante o amadurecimento neuropsicomotor normal. Essas associações indicam que as crianças mais novas, especialmente menores de cinco anos, que ainda estão em fase de aquisição das habilidades comunicativas podem apresentar manifestações transitórias que não caracterizam necessariamente, desordens fonoaudiológicas. Por outro lado, sabe-se que crianças diagnosticadas com alterações após os sete anos comumente já apresentavam indicativo de alterações em fases iniciais do desenvolvimento, o que reforça a importância de avaliações fonoaudiológicas em crianças mais novas. Além disso, observa-se que algumas crianças avaliadas apresentaram mais de uma alteração fonoaudiológica concomitante. Esse dado reitera a importância da detecção desses desvios em tempo oportuno. O diagnóstico precoce pode evitar agravamento de quadros iniciais, já que uma alteração pode estar associada à outra.^14^

Este trabalho não é isento de limitações. A primeira delas é a própria natureza transversal, sem acompanhamento longitudinal das crianças e sem inferências sobre causalidade. A impossibilidade de realização de meatoscopia e audiometria antes da avaliação do processamento auditivo e as condições ambientais para realização dos testes são também reconhecidas como limitações. Apesar disso, este estudo contribui para o avanço do conhecimento, ao abordar diferentes alterações em amostra representativa de uma população de crianças sem queixas prévias.

A alta porcentagem de crianças com alterações de linguagem oral, motricidade orofacial e processamento auditivo encontrada na população estudada pode ser indicativa da prevalência desses distúrbios em outras populações com características semelhantes. Os resultados deste estudo evidenciam a necessidade de pesquisas e ações em saúde da comunicação para o enfrentamento desse problema e reforçam ainda a premência de capacitação dos profissionais da saúde e da educação para a detecção em tempo hábil das crianças com possíveis alterações fonoaudiológicas. Espera-se que os resultados desta pesquisa possam auxiliar no planejamento de ações intersetoriais e multiprofissionais, educativas e/ou assistenciais que proporcionem oportunidades para o adequado desenvolvimento infantil e promovam de maneira global a saúde da comunicação.
